# Chronotropic incompetence in Chagas disease: effectiveness of blended sensor (volume/minute and accelerometer)

**DOI:** 10.5935/1678-9741.20150035

**Published:** 2015

**Authors:** Antonio da Silva Menezes Junior, Aline Pereira da Silva, Giovana Gurian Batista Profahl, Catarine Ottobeli, Jutay Fernando Silva Louzeiro

**Affiliations:** 1 Pontifícia Universidade Católica de Goiás (PUC-GO), Goiânia, GO, Brazil.

**Keywords:** Chagas Cardiomyopathy, Cardiac Pacing, Artificial, Heart Rate, Prospective Studies

## Abstract

**Introduction:**

Technological progress of pacemakers has allowed the association of two or more sensors in one heart rate system response. The accelerometer sensor measures the intensity of the activity; it has a relatively rapid response to the beginning of it, however, it may present insufficient response to less strenuous or of less impact exercise. The minute ventilation sensor changes the pacing rate in response to changes in respiratory frequency in relation to tidal volume, allowing responses to situations of emotional stress and low impact exercises.

**Objective:**

To evaluate the cardiorespiratory response of the accelerometer with respect to the blended sensor (BS=accelerometer sensor+minute ventilation sensor) to exercise in chagasic patients undergoing cardiopulmonary exercise test.

**Methods:**

This was a prospective, observational, randomized, cross-sectional study. Patients who met the inclusion criteria were selected. The maximum heart rate of the sensor was programmed by age (220-age). The results were analyzed through t test with paired samples (*P*<0.05).

**Results:**

Sample was comprised of 44 patients, with a mean age of 66±10.4 years, 58% were female, 54% as first implant, in 74% were functional class I and 26% were functional class II, left ventricular ejection fraction was 58±7. As for the cardiopulmonary test, maximum expected heart rate and VO2 were not achieved in both the accelerometer sensor and the blended sensor, however, metabolic equivalent in the blended sensor was higher than the expected, all data with *P*<0.001.

**Conclusion:**

Even though the maximal heart rate was not reached, the blended sensor provided a physiological electrical sequence when compared to the accelerometer sensor, providing better physical fitness test in cardiopulmonary hemodynamics and greater efficiency.

## Abstract (pt)

**Introdução:**

O progresso tecnológico dos marca passos permitiu a associação de dois ou mais sensores em um único sistema de resposta de frequência cardíaca. O sensor acelerômetro afere a intensidade da atividade, possui uma resposta relativamente rápida ao início da mesma, porém pode apresentar respostas insuficientes a exercícios físicos menos intensos ou de menor impacto. O sensor volume-minuto altera a taxa de estimulação cardíaca em resposta à variação da frequência respiratória quanto ao volume corrente, permitindo, assim, respostas a situações de estresse emocional e exercícios de baixo impacto.

**Objetivo:**

Avaliar a resposta cardiorrespiratória do acelerômetro em relação ao duplo sensor ao exercício em pacientes chagásicos submetidos ao teste ergoespirométrico.

**Métodos:**

Pesquisa prospectiva, observacional, randomizada e cruzada. Foram selecionados pacientes que preenchiam os critérios de inclusão. A frequência cardíaca máxima do sensor foi programada por idade (220-idade). Os resultados obtidos foram analisados pelo Teste T com amostras pareadas (*P*<0,05).

**Resultados:**

Amostra de 44 pacientes, com idade média de 66±10,4, com 58% do sexo feminino, 54% como primeiro implante, classe funcional I em 74% e II em 26%, fração de ejeção do ventrículo esquerdo de 58±7. Quanto ao ergoespirométrico, a frequência cardíaca máxima prevista e a VO2 máxima prevista não foram atingidas tanto no sensor acelerômetro quanto no sensor volume-minuto, contudo, o equivalente metabólico no sensor volume- minuto foi superior à prevista, todos os dados com *P*<0,001.

**Conclusão:**

Apesar da frequência cardíaca máxima não ter sido atingida, o duplo sensor proporcionou uma sequência elétrica mais fisiológica quando comparado ao sensor acelerômetro.

**Table:** 

**Abbreviations, acronyms & symbols**	
APMHR	Age-predicted maximal heart rate
BS	Blended sensor
CI	Chronotropic incompetence
GAPC	Goiás’ Arrhythmia and Pacemaker Center
HR	Heart rate
MCA	Accelerometer
MET	Metabolic equivalent
MVS	Minute ventilation sensor
PUC	Pontificai Catholic University
REC	Research Ethics Committee
TV	Tidal volume

## INTRODUCTION

Recent estimates from the World Health Organization indicate that 18 million people are infected by the *Trypanosoma cruzi*. In addition, two hundred thousand new cases happen every year. After the acute phase of the Chagas Disease, the infected individuals go through its undetermined stage, which has low morbimortality rates. Half of those will remain stable for the rest of their lives. The other half will develop a chronic form of the illness, followed by cardiac and/or digestive involvement. The chagasic cardiopathy is the most frequent chronic form of the Chagas Disease and it results in, at least, 21,000 deaths around the world every year^[1]^.

Morphological studies have shown that Chagas patients have parasympathetic denervation due to three mechanisms: *T. cruzi* direct parasitism, degeneration caused by periganglionar inflammation, and autoimmune reaction against the neurons. Besides, these patients have abnormal autonomic cardiac regulation (dysautonomia). Available studies show that it happens because of a lower sensitivity in the sinus node to the sympathetic stimulation and circulating catecholamines as well as some damage to the vagal mediated mechanism, which responds to transitory pressure changes^[2]^.

### Chronotropic Incompetence

Low tolerance to exercising in patients who have Chagas' disease may be attributed, among other causes, to chronotropic incompetence (CI), defined as the heart's inability to elevate the HR to proportionally fulfil the raise in metabolic demand^[3,4]^.

An inappropriate chronotropic response to exercising in Chagas Disease patients lowers the maximal O_2_ consumption (VO_2_ max) to between 15% and 20%. It reduces the capacity to exercise. The lack of standardization in the CI diagnostic criteria contributes to the large range of estimated prevalence in medical literature (9% to 89%). CI has been commonly diagnosed when there is failure in reaching an arbitrary percentage (85%, 80% and, less frequently, 70%) of the age-predicted maximal heart rate (APMHR), usually estimated by Astrand's formula (APMHR=220 - age±10) in the exercise stress test^[3]^.

Another variable used in the CI diagnosis is the HR reserve, which is defined as the difference between resting HR and maximal HR during graded exercise stress. When determined in percentages (adjusted HR reserve), most studies consider values lower than 80% of age-predicted heart rate reserve (APHRR)^[5]^. Therefore, four different types of CI with similar clinical repercussion are recognized: (a) failure to reach maximal HR, (b) delay in reaching maximal HR, (c) post-exercise HR inadequate recovery and (d) HR instability during exercise.

### Sensing equipment

Sensing equipment was introduced in the cardiac stimulation area as an attempt to mimic the sinus node physiological response by promoting heart rate elevation due to the raise in metabolic demand during physical exercise and emotional stress. When compared to fixed heart rate, its benefits are a result of the improvement in hemodynamic status, which is acchieved through the restoration of the cardiac debt to levels that are closer ideal, and a reduction in the arteriovenous oxygen difference. It results in performance and standard of living improvement.

In clinical practice, the most used sensing equipment are the accelerometer (ACCEL), which monitors variations in the individual's acceleration through piezoresistive or piezoelectric material, and the minute volume (MVS), which detects the thoracic impedance resulting from the raise in respiratory rate and tidal volume (TV).

The accelerometer sensor (ACCEL) measures the intensity of the activities. Moreover, it responds relatively fast in the beginning of the movements, yet it might present different insufficient responses to less intense or low impact physical exercises. The minute volume sensor (MVS) changes the cardiac stimulation ratio in response to the variation of the respiratory rate concerning the tidal volume. Hence, it allows responses to emotional stress situations and low impact exercises.

The recently obtained technological advance regarding the development of the sensing equipment^[4]^, especially the double sensors, and the poor literature on how to use them, particularly in Chagas Disease patients must be taken into account. Therefore, our study aims at evaluating the cardiorespiratory response of the accelerometer towards the double sensor (ACCEL + MVS) as far as exercising in chagasic patients who underwent the ergoespirometry test is concerned.

## METHODS

This is a forward-looking, observational, randomized and cross-sectional study, which evaluated 44 patients. They were selected according to the following criteria: presence Chagas Disease, age between 18 and 70 years old, living in Goiânia's metropolitan zone, having the sinus node disease associated to chronotropic incompetence, and using an artificial dual chamber pacemaker with two sensors, accelerometer and minute volume.

VO_2_ max represents the maximal oxygen consumption as also being the maximal amount of energy that may be produced by the aerobic metabolism in a given time unit. The energy cost in patients in the exercise stress test is given in METS, defined as metabolic equivalent, or the enough amount of energy for an individual to keep resting. When the cost is expressed in METS, we know how many times the resting metabolism was multiplied during an activity^[6]^.

Patients signed a n informed consent, and the project was approved by the Research Ethics Committee (REC) from PUC (Pontifical Catholic University)/Goiás, registered under the number 210.294/2013. Patients also underwent anamnesis, besides physical and ergoespirometry tests. The latter happened according to the Bruce protocol, on a treadmill. First, the patients remained in the biosensor ACCEL programming for 6.2±2 months and in DS for 7.1±2.5 months (r>0.05).

The biosensors programming corresponded to Astrand's formula (220 - age). Thus, they were individually programmed (named predicted rate). The patients were encouraged to exercise up to their maximal age or predicted HR (sensor-programmed).

The participants were chosen at random among those who used only one sensor or a double one and, then, they were cross-checked. Quantitative variables were described by the average and measure of dispersion (standard deviation). The obtained results were analyzed in the T Test with paired samples (r<0.05) and IBM SPSS 21 program.

## RESULTS

The 44 patients data were kept in their electronic health records, protected with a password in the Goiás Arrhythmia and Pacemaker Center (GAPC). Gender and age were analyzed as epidemiological characteristics. There was predominance of the female gender (58%) and the average age was 66±10.4 years old. Among these patients, 54% underwent the first implant, and 74% had heart failure class I and 26% had class II. The average ejection fraction from the LV through Simpson's method was 58±7%.

**Fig. 1:**
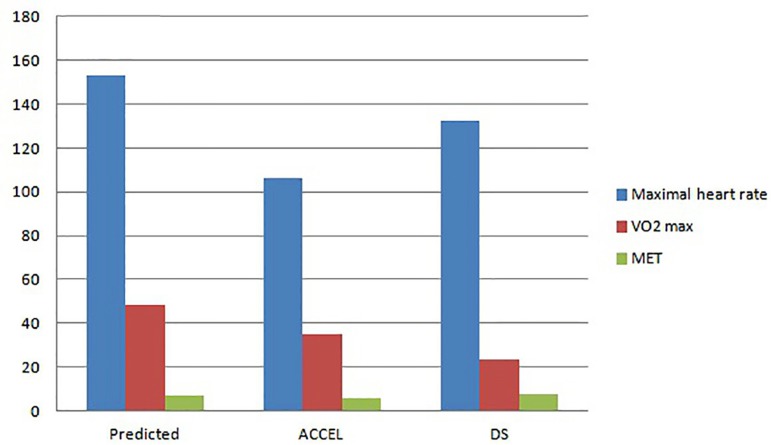
Averages obtained in the erogoespirometry test. ACCEL=Accelerometer; MET=Metabolic equivalent; DS=Double Sensor

**Fig. 2:**
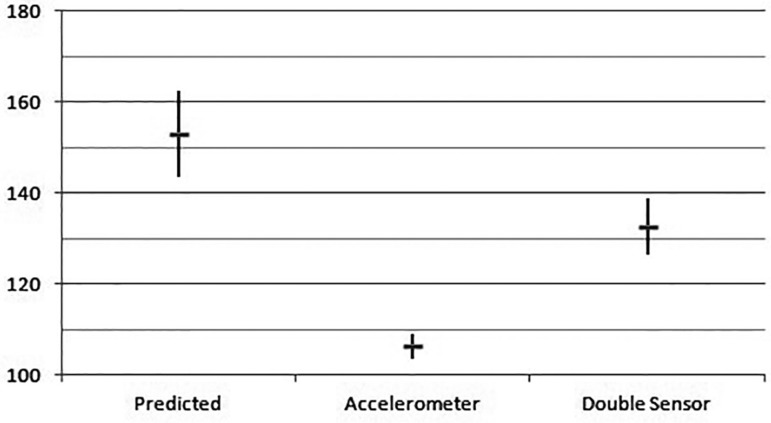
Maximal Heart Rate and standard deviation (in bpm) obtained in the ergoespirometry test.

**Fig. 3:**
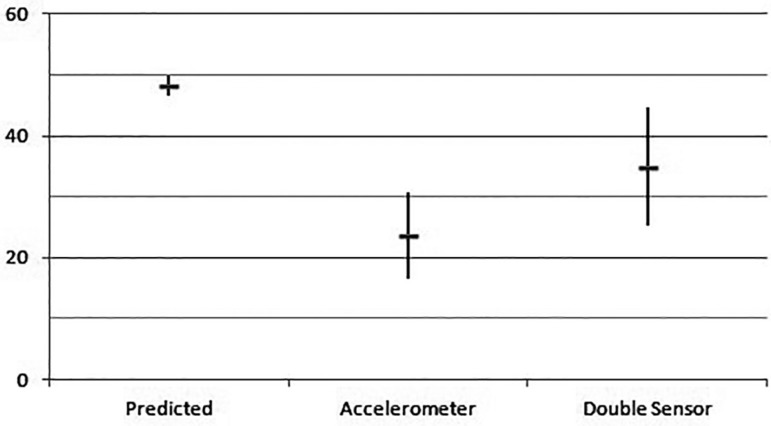
VO_2_ max: value in the ergoespirometry test in the different groups (average and standard deviation).

**Fig. 4:**
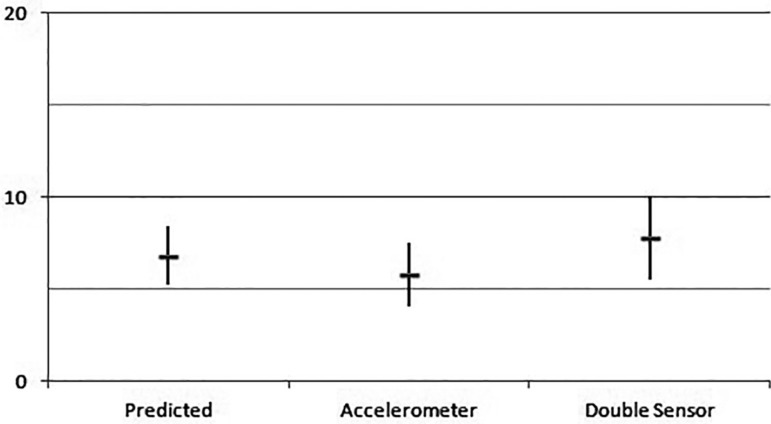
Average and standard deviation of metabolic equivalent in the different groups.

In the ergoespirometry test, the maximal predicted HR average obtained by patients was 153.0±9,4 bpm. In the patients whose pacemakers were programmed with the accelerometer, this rate reached 106.3±2.7 bpm, while the ones with double sensor had a 132.5±6.3 bpm value (r<0.0001, shown in Figure 1). As for oxygen consumption, the predicted value of VO_2_ max was 48.2±1.7. The accelerometer reached 34.9±9.7 and the double sensor reached 23.6±7.1 (r<0.0001). Eventually, the predicted metabolic equivalent (MET) reached a 6.8±1.6 value, with 5.8±1.7 METs in the accelerometer and 7.8±2.3 METs in the double sensor (r<0.0001) (Figures 2, 3 and 4).

### Arrhythmia Density Analysis

a) Atrial: there was no statistically relevant difference between the two groups (r>0.05)

b) Ventricular: there was no statistically relevant difference between the two groups (r>0.05)

## DISCUSSION

In cardiac conduction system diseases, the chronotropic pathway gets interrupted. Therefore, for the maintenance of normal physiology, there has to be installation of an artificial stimulation system, enough to supply the heart with electric disability. The great pacemakers evolution is due to their circuits and to how they adapt themselves to the metabolic needs of the patients^[6]^, even with so many types of available devices and sensors. The minute volume sensor receives the respiratory rate and it is well related to physical exercise. However, it is not completely reliable in patients with obstructive pulmonary disease and in cases of hyperventilation. The accelerometer sensor measures the body's activity or movement associated with physical activity. It is the most used sensor due to its low cost and easy programming. Nevertheless, it might not respond well in very intense exercises that involve less body movements or in emotional stress moments at rest^[7-9]^.

These data support our results. In fact, the statistically relevant difference in the metabolic equivalent (MET), comparing the ACCEL activity to the DS (ACCEL + MVS), works in a complementary way and shows great advantages and improvements in the chronotropic response. The high cost, need for closer following and reduction in the pacemaker operating life may be considered as disadvantages^[7,9]^.

In our results, none of the groups reached maximal predicted HR. That is why we cannot ascertain whether the chronotropic insufficiency is the one that leads to a lower aerobic capacity or vice-versa. In the Rocha et al.^[10]^ study, similar results were found, as far as HR is concerned. It is important to note that, after the adjustment for maximal estimated oxygen consumption, the chagasic patients went on having less aerobic capacity. It proves that the lower aerobic capacity is not the main cause of the chronotropic insufficiency observed amongst the Chagas Disease patients.

## CONCLUSION

Even though the heart rate was not reached, the double sensor provided a more physiological electric sequence compared to the accelerometer sensor.

**Table:** 

**Authors’ roles & responsibilities**	
ASMJ	Researcher
APS	Analysis and/or interpretation of data; statistical analysis; implementation of projects and/or experiments
GGBP	Performing operations and/or experiments; manuscript writing or critical review of its content
CO	Analysis and/or interpretation of data; statistical analysis; study design
JFSL	Conception and design; implementation of projects and/or experiments
